# Clinical Lecture in the Ophthalmic Department of Cook Co. Hospital

**Published:** 1874-12-15

**Authors:** F. C. Hotz


### CLINICAL LECTURE IN THE OPHTHALMIC DEPARTMENT OF THE COOK CO. HOSPITAL.

By F. C. Hotž, M.D.

*Reported by F. C. Winslow, M. D., Assistant Physician.*

GENTLEMEN: The first case we examine to-day is Hordeolum, or stye. This affection is an inflammation of one of the Meibomian glands, or of the tissues at the roots of the cilia, and is likely to occur in persons of all ages. It begins by a sense of heat in the lid, which soon becomes the seat of swelling and pain. A careful examination shows some particular point of the lid to be the seat of the pain, and at this place we find an irregular hardened nodule, which marks the position of the stye. By these signs we differentiate the disease under consideration from others which may be complicated with œdema of the lid.

These symptoms are followed in a few days by suppuration.

The treatment consists in the application of warm fomentations, and, as soon as the pus shows any tendency to point, the tumor is freely opened, the contents evacuated, and a simple dressing of lint, saturated with warm water, applied for a day or so, by which time it will usually be found to have disappeared.

CASE II, aged——.—This patient presents herself for treatment, giving the following history: Several months ago she received treatment for granular conjunctivitis, and improved to such an extent that she discontinued treatment. She says that a few days since she began to experience great pain in the head, both in the frontal and occipital region. This was accompanied by considerable febrile excitement, loss of appetite, and inability to sleep. The lids became swollen and there was a copious secretion of tears. In addition to this there is manifested great intolerance of light, the patient, as you see, constantly resorting to some means of shading the eye. The left lid is still red and tumefied, and if we examine the eye we find the conjunctiva highly congested, and exhibiting at the margin of the cornea the red zone of engorged vessels, which is characteristic of acute inflammation of the cornea and iris.

In the center of the cornea you notice an irregular yellow opacity, the surface of which is slightly elevated above the neighboring portion of the cornea. Its margin is well defined against the surrounding cloudy corneal tissue. This yellow opacity is a purulent infiltration of the cornea. The crescentic yellow opacity which you notice beneath is not in the cornea, but indicates a collection of pus in the anterior chamber—a condition known as hypopyon.

The pupil was contracted but is now slowly expanding, owing to the action of the atropine we have dropped into the eye.

The case is one of suppurative keratitis, complicated by iritis.

The affection, owing to its frequent complication of pus in the anterior chamber, is often called hypopyon keratitis.

The disease is due to a variety of causes. It may be of traumatic origin, or may follow long-continued irritation, but it is most likely to occur in persons of a scrofulous diathesis, and as a result of malnutrition.

The symptoms are those given in the history of the case before us: Deep-seated pain, intolerance of light, profuse lachrymation, swelling of the lids, dense vascularization around the cornea, yellow infiltration of a portion of the cornea, loss of sleep, and more or less febrile excitement. On the supervention of iritis we find, in addition to the above, contraction of the pupil and hypopyon.

It is impossible for us to be too guarded in our prognosis of this affection. The result depends largely upon the state of the cornea at the time of making the examination. If we find the suppuration so intense as to cause extensive destruction, we can, of course, give no encouragement to hope for a favorable result. But even in the best cases a permanent opacity will remain in the cornea, and if it be in the centre will impair the sight materially.

In the treatment of suppurative keratitis we enjoin strict rest for the affected organ. We secure this by means of the bandage, and it is necessary to exercise good judgment in the application of this simple agent, for unskillfully used it becomes a source of irritation instead of benefit. It is necessary to fill up the space between the eyebrow and nose with loose slips of cotton, which by a uniform pressure, restrain all movements of the lids over the inflamed cornea. Under the bandage we place a few layers of lint, saturated with warm water. This acts in the ordinary manner of a warm fomentation, which we like to use for all purulent inflammations. An aqueous solution of atrop. sulph. gr. ij. to ℥ j., should be freely used in the eye, where by its local sedative action it relieves the severe pain, thus contributing to the comfort of the patient. Its main object however is to combat the supervening iritis and keep the pupillary margin of the iris away from the affected part of the cornea.

The eye should be frequently examined, carefully illuminating the cornea for this purpose. As long as the margin marking the suppuration is well defined, thick and opaque, it indicates that the inflammation still continues, while on the other hand, when the margin loses its distinct outline, and the edges change from an opaque yellow to a semi-transparent gray color, it shows that the violence of the disease is abated, and the reparative stage has begun. As soon as this condition presents itself the indications for treatment are entirely different. We now hasten to evacuate the collection of pus externally, because if left to itself it might cause a perforation into the anterior chamber, followed by the sudden discharge of the aqueous humor, and the protrusion through the orifice of a portion of the iris. If, as in this case, the abscess is superficial, we merely open the anterior wall, while if it is deeply seated we perform paracentesis of the cornea, through the centre of the abscess, and thus allow the aqueous humor to escape slowly, and in its passage thoroughly washing out the pus contained in the abscess. To perform this operation you select a narrow double-edged needle, and gently introduce it through the centre of the abscess into the anterior chamber.

The subsequent treatment is simple. We maintain the organ at rest, and this, with the occasional use of the atropine, is all that is necessary.

